# Targeting miR-497-5p rescues human keratinocyte dysfunction upon skin exposure to sulfur mustard

**DOI:** 10.1038/s41419-024-06974-2

**Published:** 2024-08-10

**Authors:** Virginia Egea, Karina Lutterberg, Dirk Steinritz, Simone Rothmiller, Konrad Steinestel, Jan Caca, Andreas Nerlich, Helmut Blum, Sarah Reschke, Sajjad Khani, Alexander Bartelt, Franz Worek, Horst Thiermann, Christian Weber, Christian Ries

**Affiliations:** 1 Institute for Cardiovascular Prevention (IPEK), Ludwig-Maximilians-University (LMU) in Munich, Munich, Germany; 2 DZHK (German Centre for Cardiovascular Research), partner site Munich Heart Alliance, Munich, Germany; 3 Bundeswehr Institute of Pharmacology and Toxicology, Munich, Germany; 4 Institute of Pathology and Molecular Pathology, Bundeswehrkrankenhaus Ulm, Ulm, Germany; 5 Institute of Pathology, Academic Clinic Munich-Bogenhausen, Munich, Germany; 6 Laboratory for Functional Genome Analysis (LAFUGA), Gene Center, LMU Munich, Munich, Germany; 7 Department of Pathology, Cardiovascular Research Institute Maastricht (CARIM), Maastricht University Medical Centre, Maastricht, The Netherlands; 8 Munich Cluster for Systems Neurology (SyNergy), Munich, Germany

**Keywords:** Mechanisms of disease, Diseases

## Abstract

Sulfur mustard (SM) is a highly toxic chemical warfare agent. Exposure to SM results in various pathologies including skin lesions with subsequent impaired wound healing. To date, there are no effective treatments available. Here we discover a SM-triggered pathomechanism involving miR-497-5p and its target survivin which contributes to keratinocyte dysfunction. Transcriptome analysis using RNA-seq in normal human epidermal keratinocytes (NHEK) revealed that SM evoked differential expression of 1896 mRNAs and 25 miRNAs with many of these RNAs known to be involved in keratinocyte function and wound healing. We demonstrated that keratinocyte differentiation and proliferation were efficiently regulated by miRNAs induced in skin cells after exposure to SM. The inhibition of miR-497-5p counteracted SM-induced premature differentiation and stimulated proliferation of NHEK. In addition, we showed that microneedle-mediated transdermal application of lipid-nanoparticles containing miR-497-5p inhibitor restored survivin biosynthesis and cellular functionality upon exposure to SM using human skin biopsies. Our findings expand the current understanding of SM-associated molecular toxicology in keratinocytes and highlight miR-497-5p as feasible clinical target for specific skin therapy in SM-exposed patients and beyond.

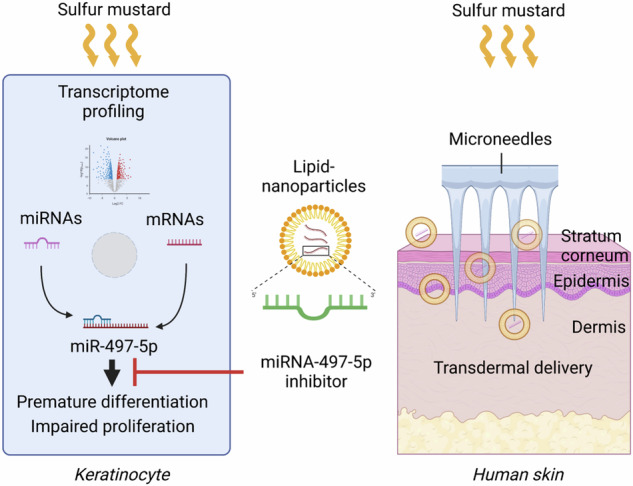

## Introduction

Sulfur mustard (SM, “mustard gas”; 2,2´-dichlorodiethyl sulfide) is a cytotoxic chemical warfare agent that has been banned by the Chemical Weapons Convention [1, 2]. Nevertheless, SM has been used repeatedly in Syria and Iraq since 2013 by state and non-state actors such as the terroristic organization “Islamic State” [3–5]. Hence, SM represents a major threat in times of war and terrorism also to civilian populations around the world. Victims of skin exposure to SM develop erythema followed by large blisters, severe burns, and display slowed healing [6, 7]. These symptoms are triggered by alkylation of cellular macromolecules including DNA and proteins [8]. However, the precise pathomechanisms in the affected skin are still unclear and call for the development of targeted therapeutic interventions [9].

Epidermal keratinocytes and dermal fibroblasts play crucial roles in the healing of skin lesions [10]. The proliferative capacity of immature keratinocytes located in the basal layer of the epidermis is pivotal in the epithelialization phase of wound healing followed by the maturation of these cells in the later stage of skin tissue remodeling [11]. Our previous studies have shown that SM affects keratinocyte growth involving dysregulation of microRNAs (miRNAs) [12, 13], but specific pathomechanisms that allow targeted therapy in SM-affected skin are still unknown.

miRNAs are a class of small non-coding RNAs and mediate post-transcriptional regulation of gene expression [14]. miRNAs bind to target mRNA transcripts and thereby induce mRNA decay and/or translational repression of protein biosynthesis. In humans, there are several hundred miRNAs which are thought to collectively regulate the majority of human genes [15]. Also, miRNAs directly and indirectly impact many regulatory pathways that control physiologic and pathological processes such as wound healing and cancer [16]. Hence, the pharmacological modulation of miRNA activity in vivo represents a promising strategy in clinical therapy [17].

Here we investigated genome-wide effects of SM on both mRNA and miRNA expression by use of high-throughput RNA sequencing (RNA-seq) technology in human keratinocytes. We identified SM-induced miRNAs and demonstrated their relevance in skin cell functions known to be essential in wound healing. This was accomplished by use of miRNA mimics and inhibitors deploying both in vitro and ex vivo studies in human skin explants. Our findings not only elaborate on the complexity of the transcriptional response to SM by epidermal cells, but also demonstrate the usefulness of miRNA inhibitors in the treatment of skin cell deficiencies.

## Material and methods

### Cultivation and treatment of cells

Primary normal human epidermal keratinocytes (NHEK) isolated from the foreskin of pooled juvenile donors were purchased from Promocell (Heidelberg, Germany). The cells were grown in serum-free keratinocyte growth medium containing 0.1 mM CaCl_2_ (low calcium conditions) (Promocell). Primary normal human dermal fibroblasts (NHDF) were purchased from Lonza (Basel, Switzerland) and cultivated in FGM-2 fibroblast growth medium containing 2% heat-inactivated fetal calf serum (FCS) and supplements provided by the distributor (Lonza). THP-1 monocytic cells were purchased from the German Collection of Microorganisms and Cell Cultures (DSMZ, Braunschweig, Germany) and grown in RPMI-1640 supplemented with 10% FCS.

Cells were maintained at 37 °C in a humidified air atmosphere in the presence of 5% CO_2_. Detachment of NHEK and NHDF was enabled by use of Accutase and trypsin/EDTA solutions from Sigma-Aldrich (Munich, Germany) and Biochrom (Berlin, Germany), respectively. For serum free conditions, NHEK were cultivated as depicted above; NHDF were grown in FGM-2, and THP-1 in RPMI-1640 supplemented with 1% Nutridoma SP (Roche Applied Biosciences, Mannheim, Germany). Cells were routinely tested for mycoplasma contamination. Cell viability was determined by trypan blue staining and periodically by application of the WST-8 assay (Dojindo, Rockville, MD, USA).

Sulfur mustard (SM; 2,2’-dichlorodiethyl sulfide; >99% purity in NMR analysis) was made available by the German Ministry of Defense. Exposure experiments were conducted by the Bundeswehr Institute of Pharmacology and Toxicology. Prior to application in each experiment, pure SM was freshly diluted in ethanol and serum-free medium to minimize hydrolysis. NHEK, native or transfected cells, were treated with the vehicle control (diluted ethanol) or with SM at final concentrations of 10 and 30 µM for 30 min at 37 °C in a specific incubator with humidified air atmosphere in the presence of 5% CO_2_. Lower dosage of SM is reported to be ineffective while higher concentrations trigger cell death in keratinocytes [13, 18, 19].

### Cell proliferation assay

Cell numbers were quantified using the CyQuant cell proliferation assay kit (Invitrogen, Darmstadt, Germany) as described previously [20].

### RNA isolation and quantitative PCR (qPCR) analysis

To study the effect of SM on the transcriptome, NHEK were cultured in the presence and absence of SM or solvent (ethanol) for 24 h at 4 replicates of each (*n* = 4). Subsequently, cells were lysed in Trizol Reagent (Thermo Fisher Scientific, Waltham, MA, USA). Total RNA was extracted from 300 µl aliquots (0.5 × 10^6^ cells) using the Maxwell® RSC miRNA Tissue Kit (Promega, Madison, WI, USA) on the Maxwell® RSC instrument according to the manufacturer’s instructions. Immediately before library construction, RNA aliquots (1 µg) were purified by treatment with Agencourt RNAClean XP Beads (Beckman Coulter, Brea, CA, USA). Quantity and integrity of RNA was determined by UV-VIS spectrometry using a Nanodrop spectrometer (Thermo Fisher Scientific) and microcapillary electrophoresis (Agilent Bioanalyzer, RNA 6000 Nano kit) (Agilent Technologies, Santa Clara, CA, USA).

Standard isolation of total RNA from NHEK, NHDF, and THP-1 cells was accomplished using the RNeasy Mini Kit (Qiagen, Hilden, Germany). On-column DNase digestion with the RNase-free DNase-set (Qiagen) was performed according to the manufacturer’s protocols. The cDNA synthesis was completed following the instructions of QuantiTect Reverse Transcription Kit (Qiagen).

qPCR was carried out on a Quantstudio 5 Real-Time-PCR-System (Thermo Fisher Scientific) using QuantiTect SYBR green PCR kit (Qiagen) according to a previously published protocol [21]. PCR primer sets and kits were applied as listed in Supplementary Table S1.

miRNA expression was determined using the miRCURY LNA PCR System (Qiagen) for conversion of RNA into cDNA and miRCURY LNA miRNA PCR Assay (Qiagen). Relative expression was normalized to the mean threshold cycle (C_T_) values of U6 that was selected for its low variability in C_T_ values during NHEK treatment. The respective primer sequences and catalog numbers are provided in Supplementary Table S1.

### RNA sequencing and data processing

For details on preparation of RNA libraries, mRNA and miRNA sequencing, data processing, and analysis see Supplementary Methods.

### Transfection of cells with miRNA mimics and inhibitors

To study miRNA function in cells, synthetic miRCURY LNA miRNAs (mimics), miRCURY LNA miRNA inhibitors, and non-specific miRCURY negative control oligoribonucleotides were applied (Qiagen). The sequences of mimics, inhibitors, and control are listed in Supplementary Table S2. Cells were transfected with 20 nM of RNA by use of Lipofectamin 2000 (Invitrogen) as described previously [20].

### Immunocytochemistry analysis and determination of Ki-67 index

To examine protein expression in cells, we deployed immunocytochemistry analysis as previously described [22]. NHEK were seeded in glass coverslip bottom chamber 8-well slides (Ibidi, Planegg, Germany) before experimental treatments. Upon completion of treatments, cells were fixed with 4% paraformaldehyde (PFA) for 15 min and permeabilized with 0.1% Triton X-100 in PBS/BSA 1% for 30 min at room temperature. The slides were subsequently incubated overnight at 4 °C with primary antibody (Table S3). Non-specific isotype antibodies were used as negative controls. Species-specific fluorescently conjugated secondary antibodies were applied for 2 h at room temperature (Table S3). The slides were embedded in Prolong^®^ Diamond antifade mountant (ThermoFisher Scientific) in the presence of 4’,6-diamidino-2-phenylindole (DAPI) to counterstain nuclei. Digital images were acquired using a Leica DMi8 fluorescence microscope equipped with a digital camera (Leica Microsystems, Wetzlar, Germany).

The protein Ki-67 was used as a marker for proliferative cells. For quantification of cells positive for Ki-67, immunofluorescence images were analyzed by ImageJ software. The Ki-67 index represents the percentage of Ki-67-positive cells within the population of DAPI-stained cells.

### Human skin model and transdermal delivery of miRNA inhibitor

Human NativeSkin® models were purchased from Genoskin (Toulouse, France). They represent 8 mm punch full thickness skin biopsies embedded in a proprietary matrix maintaining the histological features of normal skin during ex vivo culture in 12-well chambers for up to seven days; they were used as described [23]. To study the effect of SM on human skin, 10 µL of SM or ethanol as a vehicle control were applied onto the epidermal surface of freshly ordered skin explants at the indicated concentration for 30 min at 37 °C in a specific incubator and further incubated at 37 °C and 5% CO_2_ in a humidified atmosphere. The medium in the chamber containing the embedded skin biopsies was changed every day.

Transdermal delivery of miRNA was accomplished by use of 200 µl of Invivofectamine 3.0 (Invitrogen), a reagent optimized for the in vivo-application of small RNA molecules [24], containing 400 nM miRCURY LNA miRNA inhibitor (Qiagen) fluorescently labeled as indicated or negative control miRNA (Qiagen) (Table S2) applied onto the skin surface and subsequent employment of a dermapen microneedling device (Bowka, Guangdong, China) loaded with 36 Pin attachments set at 0.5 mm depth with a prick frequency of 12,000 per minute for 3 s. Delivery efficiency of fluorescently labeled miRNA inhibitor in whole skin biopsy tissues was evaluated by fluorescence imaging using Zoe Fluorescence Cell Imager (Bio-Rad, Munich, Germany) or in tissue sections using a Leica DMi8 fluorescence microscope (Leica).

### Immunohistochemistry analysis of skin biopsies

Before staining, skin biopsies were removed from their multiwell containers, fixed in 10% neutral-buffered formalin for 30 min and embedded in paraffin wax. Longitudinal cross-sections (5 µm thick) were mounted on slides and stained with hematoxylin–eosin or anti-keratin and anti-Ki-67 antibodies (Table S3), in some cases using an OptiView DAB IHC detection kit and a BenchMark Autostainer (Ventana Medical Systems, Tucson, USA). Antigen retrieval was performed in citrate buffer (113 mM citric acid, 574 mM sodium citrate tribasic dehydrate) with 0.05% Tween-20 (Sigma) and blocking by incubation in PBS containing 1% BSA and 1.25% normal horse serum for 1 h. Primary antibodies (Table S3) were incubated overnight at 4 °C in PBS containing 1% of blocking solution. Non-specific primary antibodies were used as negative controls. Fluorescently labeled secondary antibodies were used as listed (Table S3). The slides were embedded in Vectashield (Vector Laboratories) solution containing DAPI to counterstain cell nuclei. Microscopic analysis was performed by use of Leica DMi8 Fluorescence Microscope (Leica) or a Zeiss Axioscope 5 light microscope with an Axiocam 208 color camera (Zeiss, Jena, Germany). ImageJ software was applied for signal quantification.

### Gene ontology, pathway and statistical analysis

Statistical analysis of RNA-seq data was performed using R (https://www.R-project.org/). Changes of gene expression was assessed using the DESeq2 package [25]. For estimation of effect size, apeglm was used [26]. High-dose SM- (30 µM) and solvent-treated control samples (EtOH) were compared to each other. Genes with at least 2-fold change and an adjusted *P* < 0.1 were considered as differentially expressed. Heatmaps were generated using the pheatmap package (https://CRAN.R-project.org/package=pheatmap) while all other graphs from R were created with ggplot2 [27]. Biological process gene ontology (GO) annotations were used for pathway enrichment analysis for biological processes, cellular components, and molecular function. The following subsets of GO-defined genes and pathways were considered: GO0042060 (wound healing) and GO0030216 (keratinocyte differentiation). The clusterProfiler package was used for the analysis and visualization of enriched pathways [28].

Aside from RNA-seq, statistical analysis was performed using Prism 9.0 (GraphPad Software, La Jolla, CA, USA). Unless otherwise noted, descriptive statistics included mean and standard deviation or 95% Confidence Intervals (95% CI). Data distribution and homogeneity of variance were tested by the Shapiro–Wilk and Levene’s test, respectively. Comparisons between 2 groups were performed by Student’s *t* test, with Welch correction when appropriate. Comparisons among >2 groups by 1- or 2-way ANOVA with Tukey or Bonferroni post-hoc test or Kruskal–Wallis test with Dunn post-hoc test. A 2-tailed *P* < 0.05 was considered as statistically significant.

## Results

### 1894 mRNAs are differentially expressed in SM-exposed keratinocytes

**Fig. 1:**
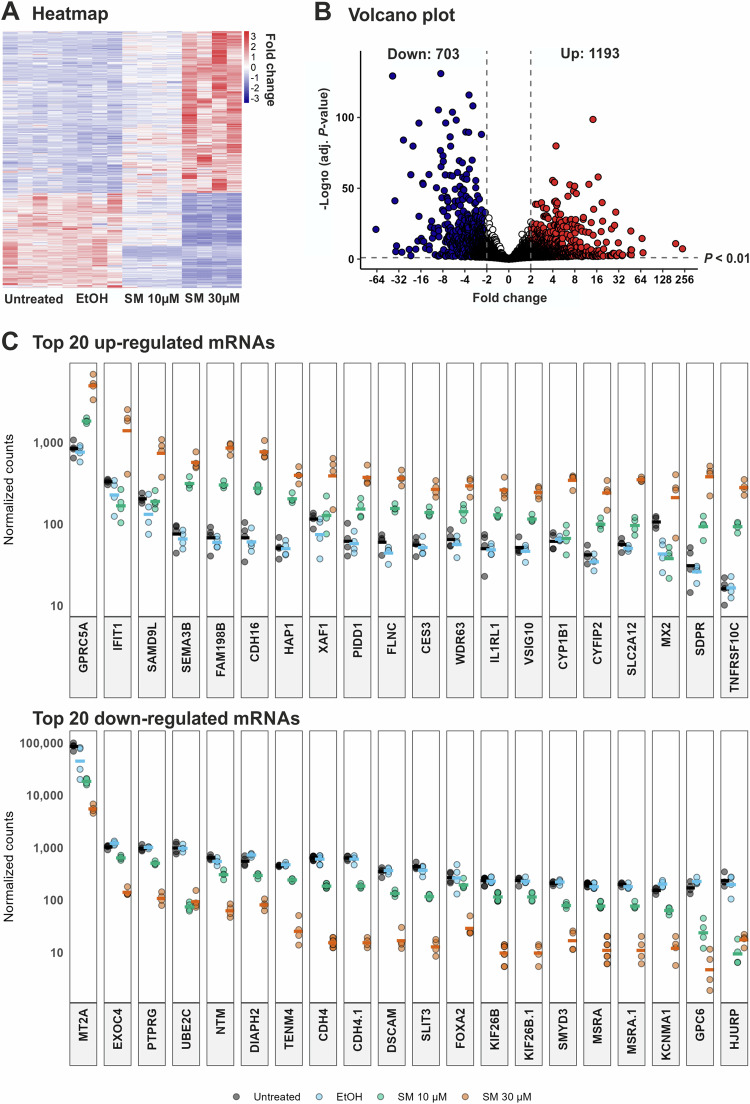
mRNA transcriptome analysis in keratinocytes upon exposure to SM. Normal human epidermal keratinocytes (NHEK) were left untreated or exposed to sulfur mustard (SM) at 10 µM and 30 µM or ethanol as vehicle control (EtOH) (*n* = 4). After 24 h of cultivation, mRNA was isolated and subjected to RNA-seq analysis. The criteria for differential expression were fold change >2 and *P* < 0.1. **A** Heatmap of the differentially expressed transcripts with red and blue color indicating up- and downregulation, respectively. Scale: relative normalized expression. **B** Volcano plot of differentially expressed mRNAs in NHEK treated with 30 µM SM in comparison to vehicle control cells. The x-axis is the fold change in mRNA expression with negative and positive values indicating downregulation and upregulation, respectively. The y-axis is the minus log_10_ scale of the adjusted *P* values indicating the levels of significance. **C** Expression levels of the top 20 upregulated and downregulated mRNAs in NHEK. Each dot represents the normalized counts for a mRNA in one group represented by a unique color. Each line represents the mean normalized counts.

To study the effect of SM on molecular processes and networks in cells, we analyzed genome-wide changes in mRNA expression in NHEK using RNA-seq (Fig. S1A). Principal component analysis (PCA) revealed clear differences in the SM-treated groups and wide overlap in the control samples that were either treated with ethanol as the solvent of SM or left untreated (Fig. S1B), suggesting dependable interpretability of the results. A total of 29,835 mRNAs have been examined. Amongst these, 1896 mRNAs were differentially expressed more than 2-fold in SM-treated NHEK in a dose-dependent manner compared to control cells treated with solvent, with 1191 mRNAs showing elevated and 703 mRNAs diminished levels of expression in SM-exposed cells (Fig. 1A, B). The top 20 mRNAs exhibiting significant up- and downregulation by SM are shown in Fig. 1C. These data indicate pronounced alterations of mRNA signatures in NHEK upon treatment with SM.

### Functional annotation and classification

**Fig. 2:**
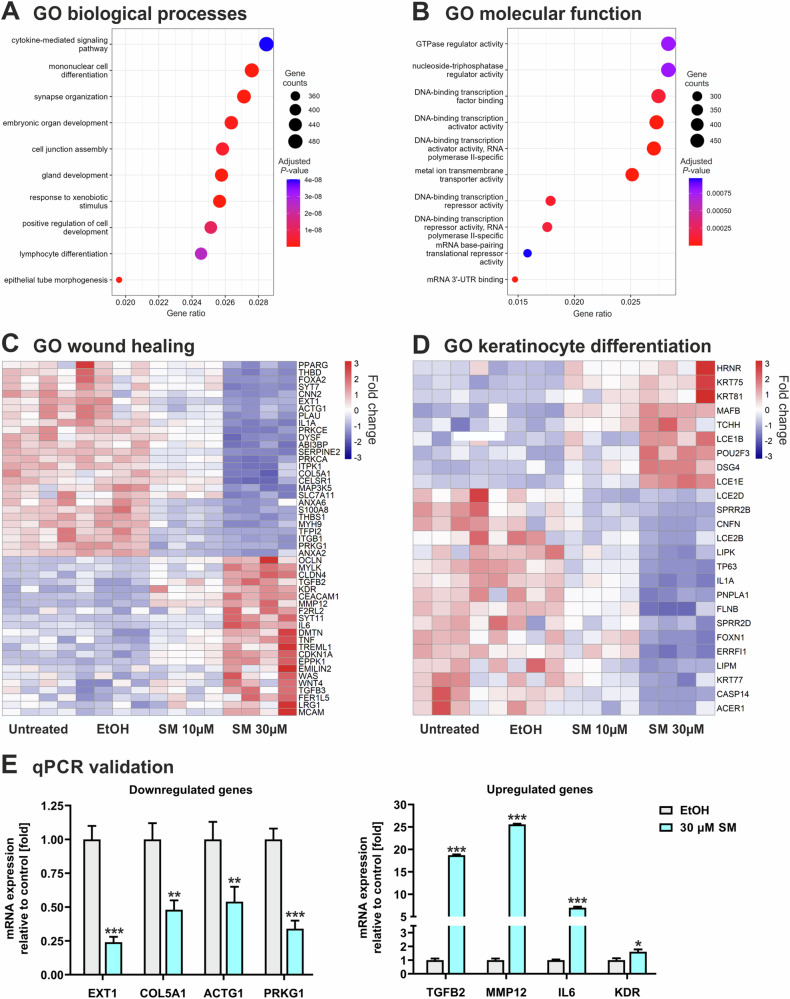
Gene ontology (GO) analyses and qPCR validation. Significantly enriched gene ontology (GO) terms in NHEK treated with 30 µM SM compared to vehicle control cells displaying the top 10 terms of biological processes (**A**) and molecular functions (**B**). The size of the circles is proportional to the number of regulated genes (*P* < 0.05) and the color of the circles reflects the Bonferroni adjusted *P* value. Heatmap of genes in GO:0042060 (wound healing) (**C**) and GO:0030216 (keratinocyte differentiation) (**D**). **E** qPCR analysis was applied to determine mRNA transcription levels of randomly selected genes employing aliquots of cDNA used for mRNA-seq analysis of NHEK treated with 30 µM SM or vehicle control (EtOH). The values were normalized to GAPDH and were given in percent to control (set 100%). All results are shown as mean values ± SD of triplicate measurements (*n* = 3). *P < 0.05, **P < 0.01, ***P < 0.001 for treated cells compared to controls as computed by Student’s unpaired *t* test.

Gene ontology (GO) analyses were made on biological processes (Fig. 2A), molecular functions (Fig. 2B), and cellular components (Fig. S1C). In biological processes, 7 of the top 10 most enriched terms were related to tissue development and cell differentiation with the greatest enhancement in cytokine-mediated signaling pathways (Fig. 2A). In molecular functions, 5 of the top 10 most enriched terms were linked to activities of DNA-binding transcription factors with the greatest enrichment in GTPase regulator activity (Fig. 2B). Cytokine activity was amongst the most enhanced terms in molecular functions (Fig. 2B) which was consistent with our results in biological processes (Fig. 2A). Moreover, mRNA 3´-UTR binding activity was significantly enriched (Fig. 2B), suggesting increased miRNA activities in SM-treated keratinocytes. The heatmap generated from genes in GO:0042060 (wound healing, 387 genes) demonstrated 22 upregulated genes, including *KDR, MYLK, TGFB2, TGFB3, IL6, TNF*, and *MMP12* as well as 27 downregulated genes such as *PPARG, COL5A, ACTG1, IL1A*, and *EXT1* (Fig. 2C). The heatmap generated from genes in GO:0030216 (keratinocyte differentiation, 156 genes) showed 6 up- and 15 downregulated genes with 7 genes belonging to the epidermal differentiation complex, namely *LCE1B, LCE1E, LCE2B, LCE2D, TCHH, SPRR2B*, and *SPRR2D*; others are also involved in keratinocyte differentiation such as *TP63, FOXN1*, and *CASP14* (Fig. 2D). The heatmap generated from genes GO:0043616 (keratinocyte proliferation, 59 genes) demonstrated 3 up- and 6 downregulated genes including *EPPK1* and *TP63*, respectively (Fig. S1D). These findings indicate that SM affects the expression of multiple genes involved in cytokine signaling and transcriptional activities involved in keratinocyte functions relevant in wound healing.

To confirm the reliability of results obtained by RNA-seq analysis, qPCR was applied to examine the expression levels of 4 randomly selected genes each downregulated (*EXT1, IL1A, COL5A1, ACTG1*) or upregulated (*TGFB2, MMP12, IL-6, MYLK*). The qPCR results were in consistency with the RNA-seq data (Fig. 2E), indicating that the RNA-seq analysis reliably identified differentially expressed genes in SM-treated keratinocytes.

### 25 miRNAs are differentially expressed in SM-exposed keratinocytes

**Fig. 3:**
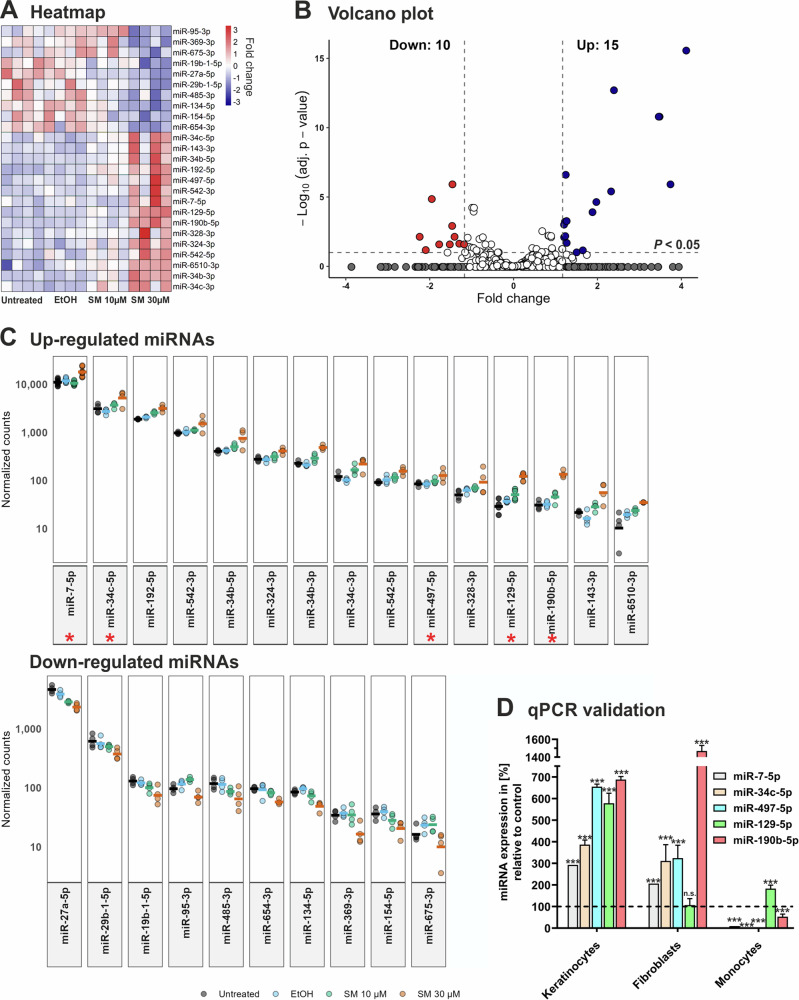
miRNA transcriptome analysis and qPCR validation. Normal human epidermal keratinocytes (NHEK) were left untreated or exposed to sulfur mustard (SM) at 10 µM and 30 µM or vehicle control (ethanol) (*n* = 4). After 24 h of cultivation, miRNA was isolated and subjected to RNA-seq analysis. The criteria for differential expression were fold change >1.5 and *P* < 0.05. **A** Heatmap of the differentially expressed miRNAs with red and blue color indicating up- and downregulation, respectively. Scale: relative normalized expression. **B** Volcano plot of differentially expressed miRNAs in NHEK treated with 30 µM SM compared to vehicle control cells. The x-axis is the fold change in miRNA expression with negative and positive values indicating downregulation and upregulation, respectively. The y-axis is the minus log10 scale of the adjusted *P* values indicating the levels of significance. **C** Expression levels of the up- and downregulated miRNAs in NHEK. Each dot represents the expression in reads per million (RPM) for a miRNA in one group represented by a unique color. Each line represents the mean of normalized counts. Red asterisks indicate miRNA candidates selected for further analysis. **D** Aliquots of cDNA used for miRNA-seq analysis of NHEK as well as cDNA from primary human fibroblasts and THP-1 monocytes treated with 30 µM SM or vehicle control (EtOH) were applied to determine miRNA transcription levels of candidate miRNAs by qPCR. The values shown were normalized to snoRNA. All results are given as mean values ± SD of triplicate measurements (*n* = 3). **P < 0.01; ***P < 0.001 for treated cells compared to controls as computed by Student’s unpaired *t* test.

RNA-seq analysis identified 25 miRNAs to be differentially expressed in NHEK upon exposure to SM with 15 miRNAs upregulated and 10 miRNAs downregulated compared to control cells treated with solvent (Fig. 3A–C). For further analysis we selected 5 candidate miRNAs from the group showing elevated expression in SM-treated NHEK (Fig. 3C), namely miR-7-5p, miR-34c-5p, miR-497-5p, miR-129-5p, and miR190b-5p. As determined by qPCR analysis, the candidate miRNA levels were significantly higher in NHEK after exposure to SM (Fig. 3D), validating our data obtained by RNA-seq. Analysis in other skin cell types revealed that treatment with SM increased the expression of 4 of the 5 candidate miRNAs in NHDF whereas in THP-1 monocytes only one of four candidate miRNAs was modulated by SM (Fig. 3D). This indicates that SM differentially impacts miRNA expression in skin cells with similar effects in epidermal keratinocytes and dermal fibroblasts.

### SM triggers differentiation and inhibits proliferation in keratinocytes

**Fig. 4:**
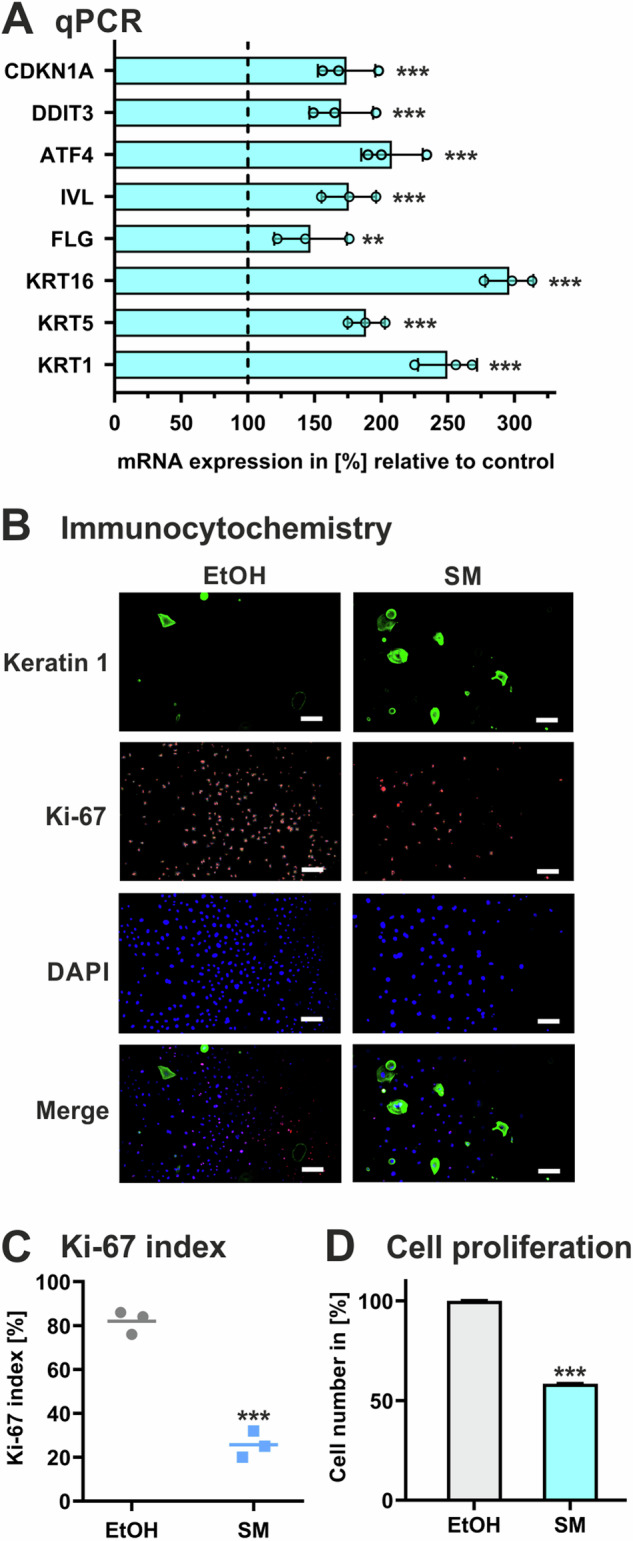
Effect of SM on differentiation and proliferation marker expression in keratinocytes. NHEK were exposed to 30 µM SM or vehicle control (EtOH). **A** After 24 h, RNA was isolated and mRNA expression levels of cyclin dependent kinase inhibitor 1 (CDKN1A), DNA damage-inducible transcript 3 protein (DDIT3), activating transcription factor 4 (ATF4), involucrin (ILV), filaggrin (FLG), keratin 16 (KRT16), keratin 5 (KRT5), and keratin 1 (KRT1) were determined by qPCR. The values were normalized to GAPDH and are given in percent to control (set 100%). **B** After 24 h of cultivation, cells were analyzed by immunocytochemistry for the expression of keratin 1 and Ki-67 stained with anti-mouse IgG (Star488, green) and anti-rabbit IgG (Star 635 P, red), respectively. DAPI (blue) was used for nuclear staining. Superimposition of keratin 1, Ki-67, and nuclear staining (merge). Magnification x 5. Scale bars, 5 µm. **C** From these data, the proliferation index was calculated by the percentage of cells positively stained for Ki-67 among the total number of cells as determined by DAPI staining using ImageJ software. **D** Quantification of cells using the CyQuant cell proliferation assay (control set 100%). Data shown in (**A**, **C**, and **D**) represent the mean ± SD of triplicate measurements (*n* = 3). Data in (**B**) are representative of 3 independent measurements (*n* = 3). **P < 0.01, ***P < 0.001 for treated cells compared to controls.

To assess the consequences of SM on cell function, NHEK were exposed to SM and first analyzed for marker expression by qPCR. mRNA levels of cyclin dependent kinase inhibitor 1, known to play a central role in the process of proliferation arrest during keratinocyte differentiation [29], was expressed higher in SM-treated NHEK in comparison to solvent-treated control cells (Fig. 4A). In addition, transcription of various factors associated with keratinocyte differentiation, such as keratin 1, keratin 5, keratin 16, filaggrin, involucrin, activating transcription factor 4, and DNA damage-inducible transcript 3 protein [29–31], were expressed higher in SM-treated NHEK (Fig. 4A). On protein level, immunocytochemistry analysis demonstrated higher levels of keratin 1 in SM-treated cells whereas Ki-67, a marker of proliferating cells [32], was significantly found at lower levels in comparison to controls (Fig. 4B, C). Concurrently, SM exposure of NHEK caused lower cell proliferation rates compared to control cells (Fig. 4D).

These findings provide evidence that SM triggers premature differentiation in keratinocytes associated with an arrest of cell proliferation.

### miRNAs control differentiation and proliferation of keratinocytes

**Fig. 5:**
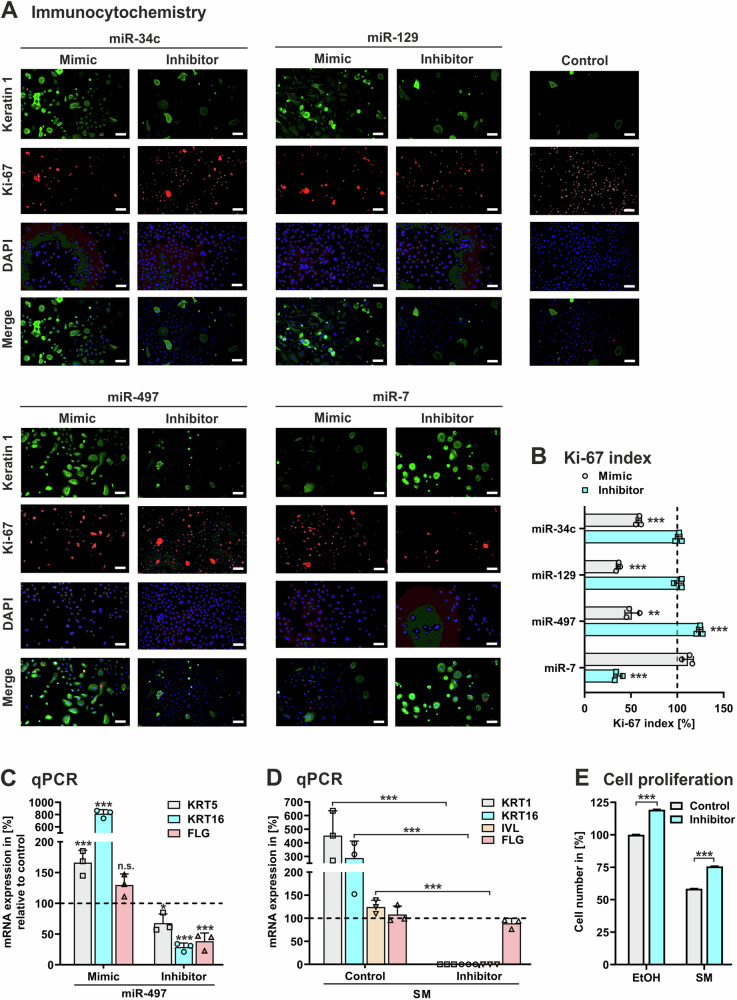
miRNA effects on differentiation and proliferation marker expression in keratinocytes. NHEK were transfected with synthetic LNA miRCURY miRNA (mimic), LNA miRCURY inhibitor of miRNA (inhibitor), or non-specific miRNA (control). **A** After 24 h of cultivation, cells were analyzed by immunocytochemistry for the expression of keratin 1 and Ki-67 stained with anti-mouse IgG (Star488, green) and anti-rabbit IgG (Star 635 P, red), respectively. DAPI (blue) was used for nuclear staining. Superimposition of keratin 1, Ki-67, and nuclear staining (merge). Magnification x 5. Scale bars, 5 µm. **B** Thereof, the proliferation index was calculated by the percentage of cells positively stained for Ki-67 among the total number of cells as determined by DAPI staining using ImageJ software. **C**–**E** NHEK transfected with miR-497-5p mimic, miR-497-5p inhibitor, or control oligonucleotides (set 100%) were treated with 30 µM SM, vehicle control (EtOH) or left untreated. After 24 h, cells were analyzed for marker expression by qPCR (**C**, **D**) and quantified using the CyQuant cell proliferation assay (**E**). Data shown in (**A**) are representative of 3 independent measurements (*n* = 3). Data in (**B**–**E**) represent the mean ± SD of triplicate measurements (*n* = 3). *P < 0.05, **P < 0.01, ***P < 0.001 for treated cells compared to controls.

We hypothesized that miRNAs might be involved in SM-mediated dysregulation of differentiation and proliferation in keratinocytes. To investigate this, we selected four candidate miRNAs that had been validated for increased expression in SM-treated NHEK, namely miR-7-5p, miR-34c-5p, miR-129-5p, and miR-497-5p (Fig. 3D). First, we analyzed the physiological roles of the candidate miRNA in keratinocyte function. Overexpression of miR-34c-5p, miR-129-5p, and miR-497-5p by transfection of NHEK with synthetic oligonucleotides (mimics) consistently caused augmentation of keratin 1 and diminishment of Ki-67 in NHEK in comparison to control cells transfected with non-specific oligonucleotides, as determined by immunocytochemistry analysis (Fig. 5A, B). The overexpression of miR-7-5p had no significant effect on keratin 1 and Ki-67 biosynthesis in NHEK (Fig. 5A, B). Notably, inhibition of miR-7-5p by transfection of the cells with complementary oligonucleotides (inhibitor) elevated keratin 1 and reduced Ki-67 while blockage of miR-34c-5p, miR-129-5p, and miR-497-5p had no such influence in NHEK (Fig. 5A, B). Very similar results by these miRNAs on Ki-67 biosynthesis were obtained in dermal fibroblasts (Fig. S2). These data indicate that increased levels of miR-34c-5p, miR-129-5p, and miR-497-5p promote differentiation and arrest proliferation in epidermal keratinocytes and dermal fibroblasts.

For further studies we focused on miR-497-5p because it is involved in the control of keratinocyte proliferation and differentiation [33]. Overexpression of miR-497-5p in NHEK significantly enhanced the mRNA levels of keratin 5, keratin 16, and filaggrin while inhibition of miR-497-5p had opposite effects as determined by qPCR analysis (Fig. 5C), suggesting miR-497-5p to promote changes in the cellular status of immature into more mature keratinocytes. Next, we investigated the role of miR-497-5p in keratinocytes upon exposure to SM. Overexpression of miR-497-5p in SM-treated NHEK further increased the mRNA levels of keratin 1, keratin 16, and involucrin compared to SM-treated control cells (Fig. 5D). Conversely, inhibition of miR-497-5p in SM-exposed NHEK completely abolished the transcription of keratin 1, keratin 16, and involucrin (Fig. 5D). These findings indicate that miR-497-5p is involved in a SM-initiated cellular maturation process by targeting factors relevant in keratinocyte differentiation. Moreover, assaying cell division revealed that inhibition of miR-497 promoted proliferation in SM-treated NHEK and in control cells, too (Fig. 5E).

Together, our data provide evidence that particular miRNAs such as miR-497-5p are potent regulators of differentiation and proliferation in epidermal cells, and suggest that inhibition of miR-497 may be useful for the intervention of SM-induced functional skin cell deficiencies.

### Transdermal delivery of miRNA inhibitor counteracts SM-induced cellular dysfunction in human skin biopsies

To assess translation of our findings from cell models into intact human skin, we employed a biopsy model of skin explants. Topical application of SM onto the epidermal surface and subsequent immunohistological analysis demonstrated that SM did not cause significant structural alterations in the epidermis 24 h after exposure but diminished Ki-67 biosynthesis in the basal keratinocytes compared to control biopsies treated with solvent (Fig. S3). These findings on Ki-67 were in consistency with our data obtained in NHEK (Fig. 4) suggesting suitability of the skin model for investigating SM-induced molecular pathomechanisms.

**Fig. 6:**
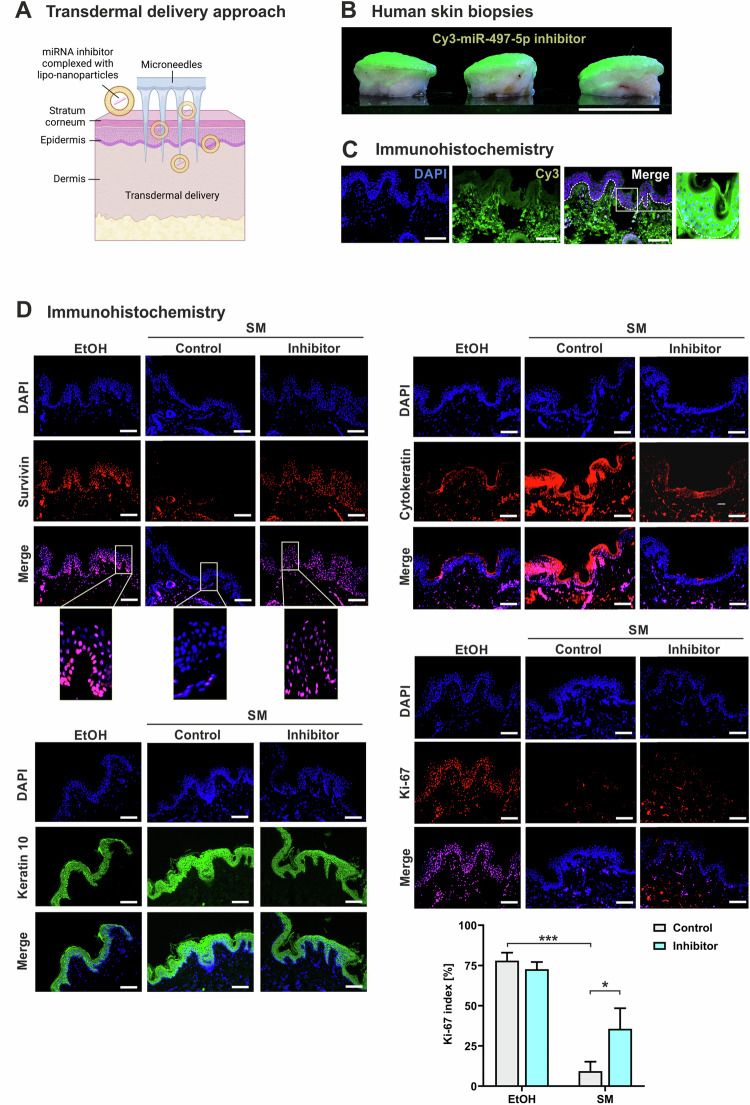
Transdermal delivery of miR-497-5p inhibitor in human skin biopsies. **A** Schematic representation of transdermal delivery of miR-497-5p inhibitor in a model of human native skin. **B**, **C** Fluorescence (Cy3)-labeled inhibitor of miR-497-5p was applied in the skin biopsies by the transdermal delivery approach. **B** Macroscopical photographs of the skin biopsies at 550 nm wavelength illumination taken after 24 h of incubation (*n* = 3). Scale bar, 11 mm. **C** Immunohistochemical analysis of cross sections of the skin biopsies. Scale bars, 100 µm. The area marked by a box has been imaged at higher magnification. **D** 10 µL 400 mM sulfur mustard (SM) or vehicle (EtOH) were added on the surface of the skin biopsies and incubated for 30 min. After transdermal delivery of miR-497-5p inhibitor or oligonucleotide controls, the skin biopsies were further cultivated for 24 h at 37 °C and 5% CO_2_ in a humidified atmosphere. Cross sections were stained with antibodies against survivin, pan cytokeratin, keratin 10, and Ki-67. Nuclear counterstaining was performed by DAPI. Superimposition of individual staining (merge). The areas marked by a box are shown in higher magnification. (*n* = 4 biopsies for each group). Scale bar, 100 µm.

Microneedles are micron-sized minimally invasive devices. They allow to bypass the stratum corneum, the topmost layer of the skin which prevents the penetration of foreign molecules into the body, providing an effective option for transdermal delivery of drugs in the treatment of various skin disorders [34–37]. To test our hypothesis that specific inhibition of miR-497-5p in epidermal and dermal skin cells might antagonize SM-induced functional disarrangements, we applied microneedling technology for transdermal delivery of miR-497-5p inhibitor complexed with lipid-nanoparticles after skin exposure to SM (Fig. 6A). Efficient and equal distribution of inhibitor into the epidermal and dermal cells of the skin explant by this approach was demonstrated by fluorescence microscopy and immunohistology analyses of fluorescence-labeled miR-497-5p inhibitor (Fig. 6B, C). Next, we confirmed the intracellular functionality of the miRNA inhibitor in the skin by analyzing a validated target of miR-497-5p and marker of immature proliferating keratinocytes, survivin [38–40]. Survivin showed highest expression levels in the basal layer of the epidermis (Fig. 6D) as to be expected. Survivin was significantly diminished in SM-exposed skin tissue but efficiently restored upon transdermal application of miR-497-5p inhibitor (Fig. 6D). Non-specific control oligonucleotides had no effect (Fig. 6D). Moreover, the transdermal administration of miR-497-5p inhibitor counterbalanced the SM-evoked increase of cytokeratins including keratin 10 and decrease of Ki-67 in the epidermis to similar levels observed in solvent-treated control cells, as demonstrated by immunohistochemical analysis (Fig. 6D).

These findings provide a proof-of-concept for the efficacy of transdermal delivery of miRNA inhibitors to compensate for molecular disarrangements in the treatment of SM-induced skin disorders.

## Discussion

Skin exposure to SM evokes formation of erythema, edema, and large blisters. These early symptoms develop into wounds with improper healing that persist for weeks or even years [41, 42]. The underlying early cellular and molecular mechanisms initiating these processes are widely unclear, and effective and targeted therapies are still unavailable. For the first time, we provide comprehensive RNA-seq data on SM-induced changes in mRNA and miRNA transcriptomes of primary human keratinocytes which serve as valuable resources for future research in the field. Derived from these results, we identified miR-497-5p and its target survivin to be involved in the arrest of proliferation and premature differentiation of keratinocytes upon exposure to SM. This pathomechanism is likely to contribute to delayed wound healing in SM-affected skin. Moreover, we established a human skin biopsy model adopting microneedling technology and lipid-nanoparticles which allowed to validate that miR-497-5p is a useful therapeutical target for the treatment of SM-evoked skin deficiencies.

Our studies revealed that the majority of genes modulated by SM in keratinocytes affect cytokine-mediated signaling pathways, cell development and differentiation as well as DNA- and RNA-binding activities. This is in agreement with previous work on SM-induced alterations in the expression of various cytokines, chemokines, and proteases in skin cells [12, 13, 43–45]. Many of these processes and regulatory factors are involved in skin inflammation and repair thereby contributing to proper wound healing. The previous finding that SM induces premature differentiation and an arrest of proliferation in keratinocytes [12] is confirmed by our results as we demonstrate that SM upregulates several factors characteristically found in more mature or wounded keratinocytes. These include keratin 1 and its binding partner keratin 10 as well as filaggrin, and involucrin, markers of terminal differentiation in keratinocytes [30], consistent with our RNA-seq data showing SM to change the expression of multiple genes relevant in keratinocyte differentiation. Keratin 16 is predominantly expressed in wounded or diseased epidermis [30, 46], agreeing with our findings of elevated keratin 16 in SM-damaged keratinocytes. Keratin 5 is typically found in immature epidermal cells [46]. Its augmentation by SM and miR-497-5p in keratinocytes suggests a different role of keratin 5 in the pathology of SM-evoked skin deficiencies. Furthermore, SM causes increase of activating transcription factor 4 as well as DNA damage-inducible transcript 3 protein, both ER-stress associated factors known to be activated during keratinocyte differentiation [31] and cyclin dependent kinase inhibitor 1, shown to be required for the cessation of proliferation during keratinocyte differentiation [29]. Thus, arrest of proliferation accompanied by premature maturation seems to be a functional hallmark in SM-affected keratinocytes. Importantly, physiological differentiation of keratinocytes in the epidermis is strictly regulated in terms of space and time [30, 46], while SM causes this process to be rather uncoordinated.

Through their targeting of mRNA translation, miRNAs are potent regulators of cellular functionality. Our findings on differential expression of miRNAs in SM-exposed keratinocytes is in line with previous work reporting on changes of miRNA levels in primary human skin cells, cell lines, animal models, as well as patient´s skin upon exposure to SM [13, 47–49]. The observation that augmentation of miR-34c-5p, miR-129-5p, and miR-497-5p consistently promoted differentiation and arrested proliferation in epidermal keratinocytes and dermal fibroblasts while miR-7-5p did not, highlights the relevance of miRNAs in the control of downstream regulatory networks and cellular effects which probably contribute to SM-evoked skin pathophysiology. In agreement with our data, miR-34c blocks proliferation in epidermal cells [50]. This is especially interesting because keratinocytes isolated from non-healing wounds express elevated levels of miR-34c promoting the production of proinflammatory cytokines [51], similar to SM-affected keratinocytes [52]. On the other hand, miR-129 seems to play a beneficial role in non-healing diabetic wounds [53]. miR-497-5p reportedly promotes differentiation and inhibits proliferation in the process of epithelial to mesenchymal transition of keratinocytes [33]. This is in agreement with the effect of miR-497-5p in these cells after exposure to SM. Our observation that inhibition of particular miRNAs reconstituted differentiation and proliferation in SM-affected keratinocytes supports the idea that miRNAs might be valuable targets for clinical intervention of SM-evoked dysfunctions in skin cells.

To test this hypothesis, we used human skin biopsies [54]. Our findings that the intradermal discharge of miR-497-5p inhibitor into SM-treated skin alleviated epidermal dysfunctions are in line with encouraging results obtained by similar miRNA-based strategies for clinical intervention of various skin disorders including skin cancer and (chronic) wound healing [55, 56]. Survivin/BIRC5 is a validated target of miR-497-5p [38, 39] as reflected by survivin´s downregulation in SM-exposed keratinocytes. In the epidermis, survivin controls cell growth by high expression in the nucleus of immature proliferating keratinocytes of the basal layer, but decreasing levels during differentiation of these cells when forming the upper layers of the epidermis [40]. The fact that survivin depletion in SM-affected skin cells was restored by the application of miR-497-5p inhibitor proves the efficacy of our transdermal delivery approach.

One limitation of our studies is that we considered only one time point because we focused on early molecular effects that initiate subsequent functional changes in the epidermis. Keratinocyte proliferation and differentiation, especially during wound healing, are highly dynamic processes with complex transcriptional alterations and cellular effects developing over a long period of time. Hence, time course experiments are necessary to allow a deeper insight into the pathomechanisms leading to SM-evoked short-term effects such as erythema and blister formation which later-on develop into delayed wound healing. Animal trials would be necessary to comprehensively investigate all aspects of physiological wound healing, including blood flow-associated edema formation and immune cell recruitment into affected skin tissues. However, animal experiments using chemical warfare agents must be designated as incriminatory animal testing which is not permissible in Germany. Certainly, deciphering relevant mRNA targets and their downstream pathways affected by SM-modulated miRNAs in a broader spectrum of cells including inflammatory cells, fibroblasts, and endothelial cells would provide further important information to enhance the understanding of SM action in the skin. Notably, miRNAs not only repress but also stimulate the expression of specific target genes by direct and indirect mechanisms [57, 58] enhancing the complexity of downstream-regulatory networks. Nevertheless, our RNA-seq results in epidermal cells captured the effects of SM on the expression of hundreds of genes painting a detailed molecular picture of both mRNA and miRNA changes that can be starting points for further investigations. Moreover, the results of our experimental studies highlight the suitability of miRNAs including miR-497 as valuable candidates for a targeted therapy aiming to improve re-epithelialization and wound healing in skin lesions of patients after exposure to SM.

### Supplementary information

Supplementary Figures and Legends

Supplementary Tables

Supplementary Methods

Checklist AJ

## Data Availability

The RNA sequencing data from this publication have been deposited to the GEO repository database and assigned the identifier GSE239682. Other datasets are available from the corresponding author on reasonable request.
