# Supplementary material for: Targeting miR-497-5p rescues human keratinocyte dysfunction upon skin exposure to sulfur mustard
Source: Cell Death Dis. 2024 Aug 10;15(8):585. doi: 10.1038/s41419-024-06974-2 (PMC11316827; doi:10.1038/s41419-024-06974-2)
Supplement: Supplementary file 1 — Supplementary Figures and Legends [file 41419_2024_6974_MOESM1_ESM.docx]

# Supplementary FIGURES AND FIGURE LEGENDS

**Supplementary figure S1**


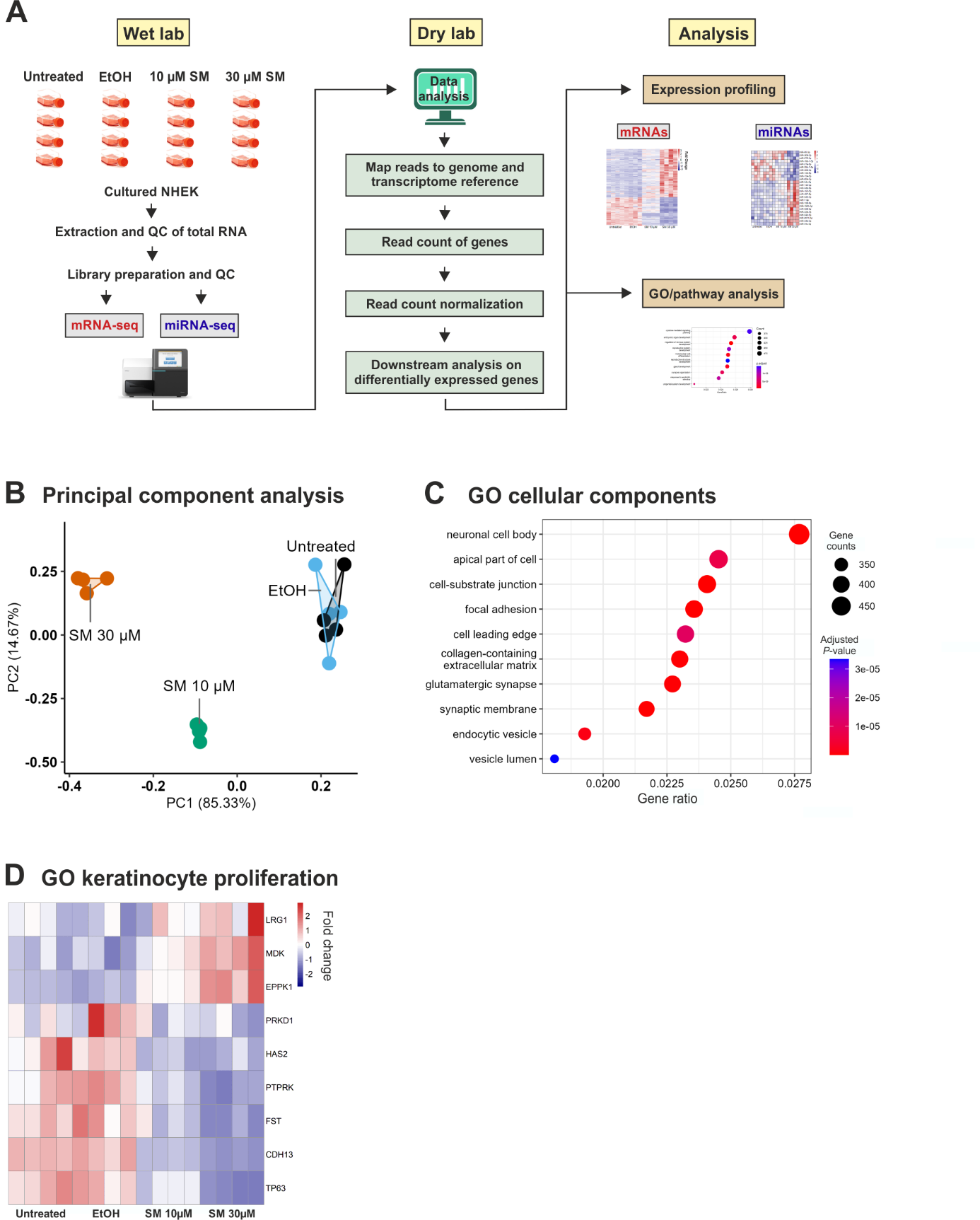


**Fig. S1. Transcriptome and GO pathway analysis in NHEK upon exposure to SM.** **(A)** Workflow scheme of RNA-sequencing and subsequent data analyses. Normal human epidermal keratinocytes (NHEK) were left untreated or exposed to sulfur mustard (SM) at 10 µM and 30 µM or ethanol as vehicle control (EtOH) (n = 4). After 24 hours of cultivation, mRNA or miRNA was isolated and used to construct mRNA- or miRNA-libraries. After sequencing, differentially expressed mRNAs or miRNAs were identified by comparative analysis of mRNA and miRNA profiles. **(B)** Principal component analysis of the mRNA transcriptome. **(C,D)** Significantly enriched gene ontology (GO) terms in NHEK treated with 30 µM SM compared to vehicle control cells displaying the top 10 terms of cellular components (C) and keratinocyte proliferation (D). The size of the circles is proportional to the number of regulated genes (*P* < 0.05) and the color of the circles reflects the Bonferroni adjusted *P* value.

**Supplementary figure S2**


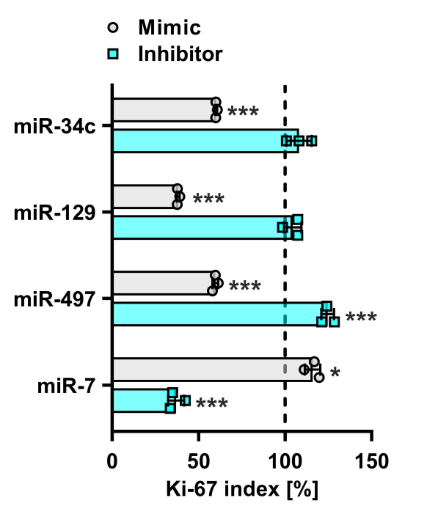


**Fig. S2. miRNA effects on proliferation marker expression in dermal fibroblasts.** Cells were transfected with synthetic LNA miRCURY miRNA (mimic), LNA miRCURY inhibitor of miRNA (inhibitor), or non-specific miRNA (control). After 24 hours of cultivation, cells were analyzed by immunocytochemistry for the expression of Ki-67 stained with anti-mouse IgG (Star488, green) and anti-rabbit IgG (Star 635P, red), respectively. DAPI (blue) was used for nuclear staining. Thereof, the proliferation index was calculated by the percentage of cells positively stained for Ki-67 among the total number of cells as determined by DAPI staining using ImageJ software. Data shown represent the mean ± SD of triplicate measurements (*n* = 3). **P* < 0.05, ****P* < 0.001 for cells transfected with mimic or inhibitor compared to controls.

**Supplementary figure S3**


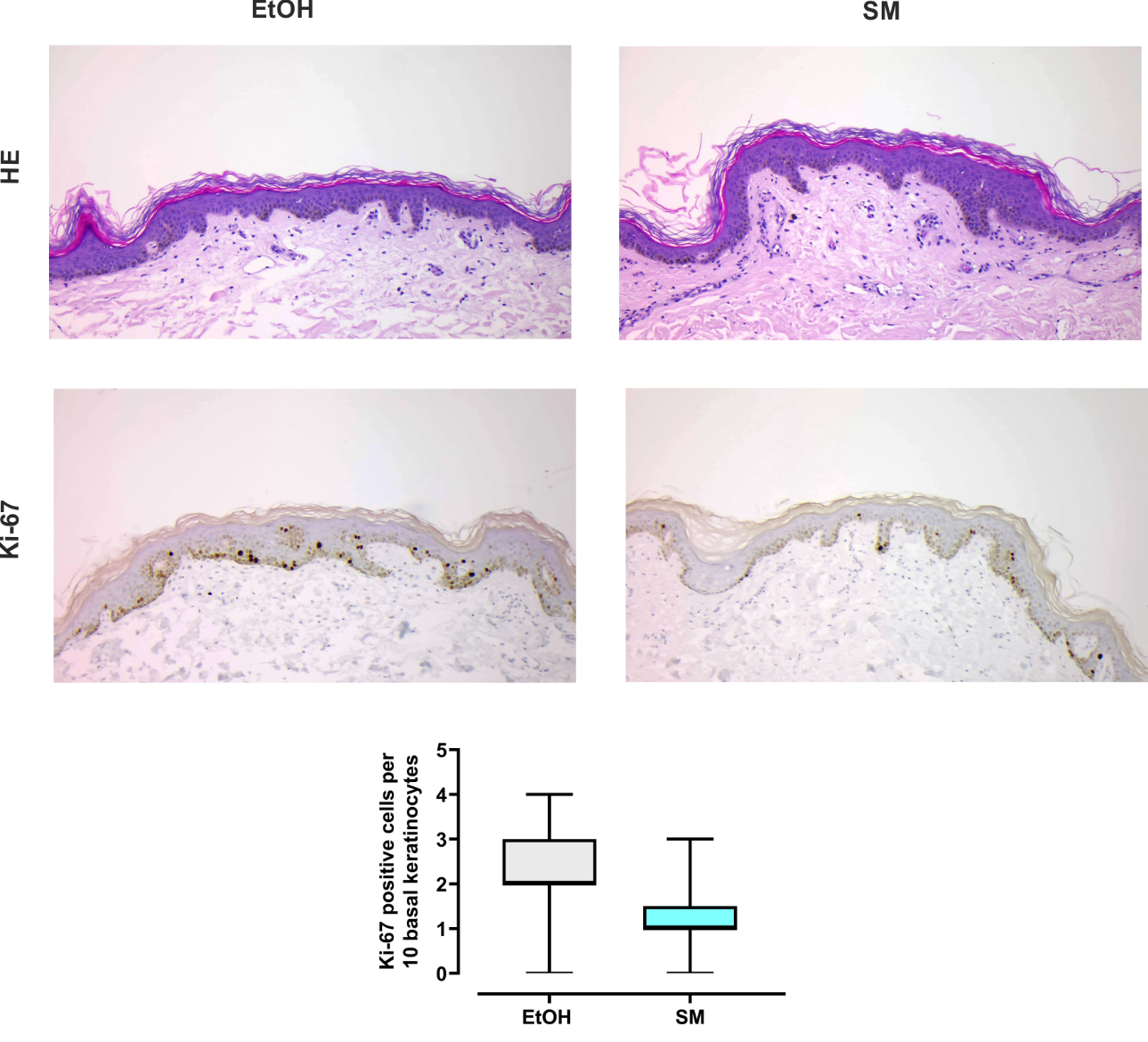


**Fig. S3. Immunohistochemistry analysis of human skin biopsies upon exposure to SM.**

10 µL of 8 M sulfur mustard (SM) or ethanol as vehicle control (EtOH) were added onto the surface of a model of native human skin and incubated for 30 minutes. After further cultivation at 37°C and 5% CO_2_ in a humidified atmosphere for 1 and 3 days, cross sections were stained with hematoxylin-eosin (HE) and antibodies against Ki-67. Magnification 20x. Three replicates were used for each treatment (*n* = 3 biopsies per group).

**Supplementary figure S4**


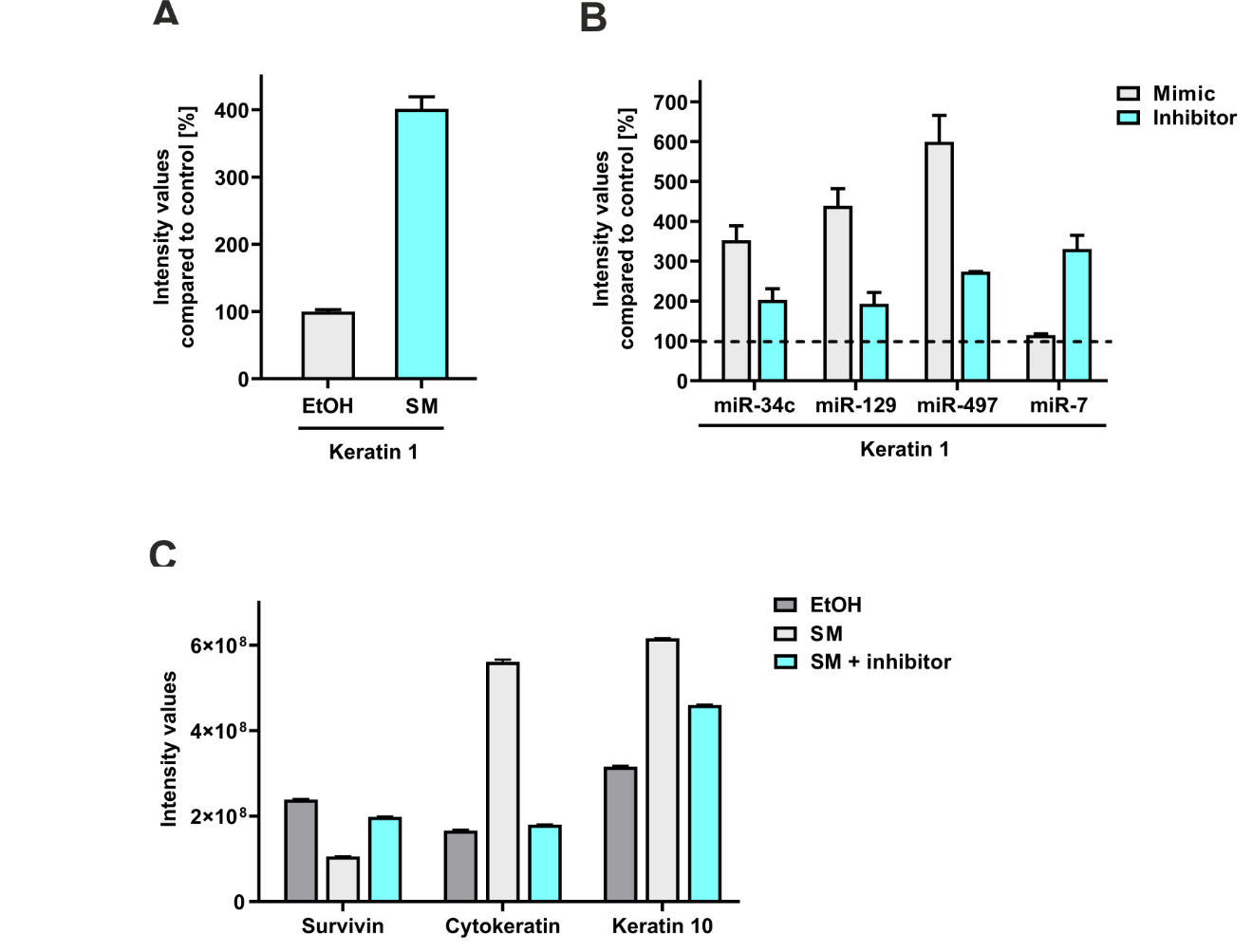


**Fig. S4. Semiquantitative analysis of immunocytochemical and immunohistochemical stainings.**

Fluorescence intensities were calculated using ImageJ software. A) Keratin 1 biosynthesis was increased in keratinocytes upon treatment with SM as shown in Figure 4B. B) Keratin 1 biosynthesis was differentially regulated by miRNA and miRNA inhibitors as shown in Figure 5A. C) The SM-induced diminishment of survivin and augmentation of cytokeratin as well as keratin 10 were efficiently counterbalanced upon transdermal delivery of inhibitor of miR-497-5p in the epidermis of human skin biopsies as shown in Figure 6D. Sulfur mustard (SM); ethanol as vehicle control (EtOH); the fluorescence intensity was determined in three different areas of each image (*n* = 3).
