# Supplementary material for: Targeting miR-497-5p rescues human keratinocyte dysfunction upon skin exposure to sulfur mustard
Source: Cell Death Dis. 2024 Aug 10;15(8):585. doi: 10.1038/s41419-024-06974-2 (PMC11316827; doi:10.1038/s41419-024-06974-2)
Supplement: Supplementary file 2 — Supplementary Tables [file 41419_2024_6974_MOESM2_ESM.docx]

# Supplementary Tables

**Table S1. Sequence of qPCR primer sets.**

| **Hsa transcript** | **Forward** | **Reverse** |
| --- | --- | --- |
| EXT1 | GGCAAAAGCACAAGGATTCTCGC | CTGCAAAGCCTCCAGGAATCTG |
| COL5A1 | GCCCGGATGTCGCTTACAG | AAATGCAGACGCAGGGTACAG |
| ACTG1 | CATTGTCATGGACTCTGGAGAC | GAGGATCTTCATGAGGTAGTC |
| PRKG1 | GACAACGATGAACCACCAC | GCTTTGCTTCAGGACCAC |
| TGFB2 | CAGCACACTCGATATGGACCA | CCTCGGGCTCAGGATAGTCT |
| MMP12 | CATGAACCGTGAGGATGTTGA | GCATGGGCTAGGATTCCACC |
| IL6 | ACTCACCTCTTCAGAACGAATTG | CCATCTTTGGAAGGTTCAGGTTG |
| KDR | GGAACCTCACTATCCGCAGAGT | CCAAGTTCGTCTTTTCCTGGGC |
| CDKN1A | TCAGGGTCGAAAACGGCGGC | TTTGAGGCCCTCGCCGCTTCC |
| DDIT3 | AAAGATGAGCGGGTGGCAGCG | AGCTGCCATCTCTGCAGTTGGAT |
| ATF4 | TCAGGGTCCACGGCCACCAT | ACGCTGCTGCTGAATGCCGT |
| IVL | CTGCCTCAGCCTTACTGTGA | GGAGGAGGAACAGTCTTGAGG |
| FLG | CTGGACACTCAGGTTCCCAT | TTTCGTGTTTGTCTGCTTGC |
| KRT16 | TGCCCACCTTTCCTCCCAGCAA | CCGGGTCTGACGGCTCGAAG |
| KRT5 | CTGCTGGAGGGCGAGGAATGC | CCACCGAGGCCACCGCCATA |
| KRT1 | ATTTCTGAGCTGAATCGTGTGATC | CTTGGCATCCTTGAGGGCA |
| GAPDH | Qiagen QuantiTect Primer Assay cat. no. QT02504278 | |

Hsa, Homo sapiens.

**Table S2. miRNA mimics, inhibitors, qPCR kits, and controls.**

| **Hsa transcript** | **Sequence / supplier / catalogue / number** |
| --- | --- |
| miR-7-5p mimic | Qiagen miScript MIMIC cat. no. MSY0000067 |
| miR-7-5p inhibitor | Qiagen MiRCURY LNA INHIBITOR cat. no. MIN0000067-ADA |
| miR-7-5p qPCR | Qiagen MiRCURY LNA miRNA PCR cat. no YP00205877 |
| miR-34c-5p mimic | Qiagen LNA MIMIC cat. no. YM00473212 |
| miR-34c-5p inhibitor | Qiagen MiRCURY LNA INHIBITOR cat. no. YM00473212-ADA |
| miR-34c-5p qPCR | Qiagen MiRCURY LNA miRNA PCR cat. no YP00205659 |
| miR-129-5p mimic | Qiagen miScript MIMIC cat. no. MSY0000242 |
| miR-129-5p inhibitor | Qiagen MiRCURY LNA INHIBITOR cat. no. Y104100088-ADA |
| miR-129-5p qPCR | Qiagen MiRCURY LNA miRNA PCR cat. no. YP00204534 |
| miR-190b-5p mimic | Qiagen LNA MIMIC cat. no. YP00206031 |
| miR-190b-5p inhibitor | Qiagen MiRCURY LNA INHIBITOR cat. no. YP00206031-ADA |
| miR-190b-5p qPCR | Qiagen MiRCURY LNA miRNA PCR cat. no YP00206031 |
| miR-497-5p mimic | Qiagen miScript MIMIC cat. no. MSY0002820 |
| miR-497-5p inhibitor | Qiagen MiRCURY LNA INHIBITOR cat. no. Y104101348-ADA |
| miR-497-5p qPCR | Qiagen MiRCURY LNA miRNA PCR cat. no YP00204354 |
| Inhibitor control | miRCURY LNA miRNA Power Inhibitor Control. cat.no 339130 |
| Oligonucleotide control | Qiagen AllStars Negative Control siRNA cat. no. 1027280 |
| Snord61 | Qiagen miScript cat. no. MS00033705 |
| Snord72 | Qiagen miScript cat. no. MS00033719 |
| U6 | Qiagen QuantiTect Primer Assay cat. no. YP02119464 |
| U2 | Qiagen QuantiTect Primer Assay cat. no. QT00097594 |
| GAPDH | Qiagen QuantiTect Primer Assay cat. no. QT02504278 |

Hsa, Homo sapiens.

**Table S3.** List of antibodies.

| **Antigen** | **Supplier** | **Clone /**  **Cat. no.** | **Concentration** | |
| --- | --- | --- | --- | --- |
|  |  |  | **IC** | **IHC** |
| Keratin 1 | Biolegend | PRB-149P-100 | 1:300 | - |
| Keratin 10 | Chemicon | mab3230 | - | 1:25 |
| Pan cytokeratin | Abcam | ab7753 | - | 1:25 |
| Ki-67 | Invitrogen | 14-5699-82 | 1:250 | 1:25 |
| Ki-67 | Zytomed | K-2 | - | Undiluted |
| Survivin | Abcam | ab469 | - | 1:25 |
| Goat anti-mouse IgG (Star488) | Abberior | ST488-1001 | 1:500 | - |
| Goat anti-rabbit IgG (Star635P) | Abberior | ST635P-1002 | 1:500 | - |

IC, immunocytochemistry; IHC, immunohistochemistry; Cat. no., catalogue number
