# Supplementary material for: Targeting miR-497-5p rescues human keratinocyte dysfunction upon skin exposure to sulfur mustard
Source: Cell Death Dis. 2024 Aug 10;15(8):585. doi: 10.1038/s41419-024-06974-2 (PMC11316827; doi:10.1038/s41419-024-06974-2)
Supplement: Supplementary file 3 — Supplementary Methods [file 41419_2024_6974_MOESM3_ESM.docx]

**SUPPLEMENTARY METHODS**

**Preparation of RNA libraries and RNA sequencing**

A total of 32 RNA-seq libraries were prepared from samples that underwent SM incubation for 24 hours, consisting of 16 mRNA libraries and 16 miRNA libraries. To ensure reproducible results despite labor-intensive procedures, mRNA as well as miRNA libraries were constructed from two batches of randomly selected samples.

Sequencing libraries were constructed from poly-A RNA with the SENSE mRNA-Seq Library Prep Kit V2 (Lexogen, Wien Austria) essentially according to the manufacturer’s instructions. In brief, 300 ng of total RNA (RNA integrity number (RIN) > 8.0) were processed with half of the reaction volume described in the manual. The PCR products were purified with Agencourt AMPure XP beads (Beckman Coulter). Library quality was validated using an Agilent Bioanalyzer (DNA 1,000 Kit). Barcoded libraries were pooled and sequenced on an Illumina HiSeq1500 as 50-nt single-end reads with an average sequencing depth of 20 million reads per sample.

miRNA sequencing libraries were constructed using the QIAseq® miRNA Library Kit (Qiagen) starting with 100 ng of total RNA following the manufacturer’s instructions. Finally, amplified miRNAs libraries were purified using Agencourt AMPure XP beads (Beckman Coulter, #A63880). The quality of the libraries was assessed by microcapillary electrophoresis (Agilent Bioanalyzer, DNA 1,000 Kit). The finished libraries were pooled and sequenced on an Illumina HiSeq1500 as 50-nt single-end reads with an average sequencing depth of 30 million reads per sample.

**RNA sequencing data processing and analysis.**

mRNA and miRNA sequencing data were preprocessed on a Galaxy server at the Laboratory for Functional Genome Analysis (LAFUGA), Gene Center, LMU Munich, Germany ([Afgan, Baker et al., 2016](#_ENREF_1)). The mRNA and miRNA datasets were demultiplexed by a inhouse demultiplexer. Adapter sequences were removed from the 3’-end using Clip adaptor sequence (version 1.0.0, tggaattc, seed 8, mismatch 0, minimum length after clipping 1). For mRNA data, 9 nucleotides caused by random priming on the 5’-end of the reads were trimmed using FastqFilter (version 1.0.). Reads passing the filtering were aligned to the human genome (hg38 from GENCODE) with STAR (version 2.4.1b, default options: up to 4% mismatches and no multimapping allowed) ([Dobin, Davis et al., 2013](#_ENREF_3)). Reads per gene were counted using htseq-count (version 1.0.0) ([Anders, Pyl et al., 2015](#_ENREF_2)) with default settings and GENCODE gene annotation (hg38). Differentially expressed genes between SM treated and control samples were detected using DESeq2 (Version 2.11.40.7, significance cutoff, adjusted P value < 0.01, Benjamini-Hochberg correction) ([Love, Huber et al., 2014](#_ENREF_6)).

After demultiplexing and adaptor trimming, the miRNA reads were mapped to the human genome (hg38 from GENCODE) using MiRDeep2 mapper (Version 2.0.0) ([Friedländer, Mackowiak et al., 2012](#_ENREF_4)) and the abundant reads were quantified using MiRDeep2 quantifier (Version 2.0.0) ([Mackowiak, 2011](#_ENREF_7)). Differential gene expression analysis was performed using DESeq2 (Version 2.11.40.6, FDR < 0.05) ([Friedländer et al., 2012](#_ENREF_4)).

**mRNA-seq quality metrics and complexity analysis**

Coding genes were captured by polyA-specific libraries constructed from total RNA after a 24 h treatment of NHEK with 10 µM and 30 µM SM or solvent (ethanol) and left untreated as controls. The experiments were performed with four biological replicates. Libraries were sequenced at a depth of ~30 million reads per sample. After mapping of sequence reads to the human reference genome (hg38), RNA-Seq specific quality metrics were assessed, including alignment rate and percentage of rRNA (Table S1). The majority of sequence reads were uniquely aligned (>76% uniquely mapped reads) to the reference genome (hg38) and captured 12,500-15,000 genes by at least 10 reads. The numbers of reads per library and detected genes are reported in Supplementary Table S2. To identify genes that are differentially abundant between SM-treated and control NHEK samples a negative binomial generalized linear model was used (DESeq2) ([Love et al., 2014](#_ENREF_6)).

Table S1: Quality metrics for mRNA-seq. Results from the STAR (Version 2.4.1b) and SortMeRNA (Version 2.1b.6) ([Kopylova, Noé et al., 2012](#_ENREF_5)) modules are provided. The quality metrics of mRNA-Seq are shown, which include the count of sequenced reads, the percentages of reads uniquely mapped to the reference genome, and reads mapped to rRNA.

| **Sample_ID** | **Group assignment** | **Number of input reads** | **Uniquely mapped reads [%]** | **rRNA reads [%]** |
| --- | --- | --- | --- | --- |
| Control 5A | 24h-untreated | 2,24E+07 | 75.81% | 2.57% |
| Control 5B | 24h-untreated | 2,72E+07 | 76.90% | 2.09% |
| Control 5C | 24h-untreated | 1,91E+07 | 75.45% | 2.65% |
| Control 5D | 24h-untreated | 1,92E+07 | 75.69% | 3.64% |
| EthOH 6A | 24h-EtOH 6A | 2,03E+07 | 76.17% | 3.22% |
| EthOH 6B | 24h-EtOH 6B | 2,36E+07 | 75.81% | 3.00% |
| EthOH 6C | 24h-EtOH 6C | 3,24E+07 | 72.49% | 8.30% |
| EthOH 6D | 24h-EtOH 6D | 3,45E+07 | 76.10% | 2.10% |
| 10 µM SM 7A | 24h-10 µM SM 7A | 2,31E+07 | 76.05% | 3.45% |
| 10 µM SM 7B | 24h-10 µM SM 7B | 2,51E+07 | 75.80% | 3.57% |
| 10 µM SM 7C | 24h-10 µM SM 7C | 3,17E+07 | 76.33% | 2.90% |
| 10 µM SM 7D | 24h-10 µM SM 7D | 2,92E+07 | 76.27% | 2.84% |
| 30 µM SM 8A | 24h-30 µM SM 8A | 2,64E+07 | 75.75% | 3.79% |
| 30 µM SM 8B | 24h-30 µM SM 8B | 3,15E+07 | 76.15% | 4.41% |
| 30 µM SM 8C | 24h-30 µM SM 8C | 3,28E+07 | 75.92% | 4.44% |
| 30 µM SM 8D | 24h-30 µM SM 8D | 2,75E+07 | 71.55% | 9.71% |

Table S2: Library Complexity Analysis of mRNA-seq Data. The polyA-specific libraries'-sequence data were aligned to the human hg38 reference genome using STAR (version 2.7.6a). HTSeq-Counts results were use to determine the number of genes covered by at least 10 sequence reads.

| **Sample_ID** | **Group assignment** | **Number of input reads** | **Number of detected genes (>10 reads)** |
| --- | --- | --- | --- |
| Control 5A | 24h-untreated | 2,24E+07 | 12.919 |
| Control 5B | 24h-untreated | 2,72E+07 | 13.112 |
| Control 5C | 24h-untreated | 1,91E+07 | 12.589 |
| Control 5D | 24h-untreated | 1,92E+07 | 12.680 |
| EthOH 6A | 24h-EtOH 6A | 2,03E+07 | 12.490 |
| EthOH 6B | 24h-EtOH 6B | 2,36E+07 | 12.777 |
| EthOH 6C | 24h-EtOH 6C | 3,24E+07 | 13.179 |
| EthOH 6D | 24h-EtOH 6D | 3,45E+07 | 13.337 |
| 10 µM SM 7A | 24h-10 µM SM 7A | 2,31E+07 | 13.034 |
| 10 µM SM 7B | 24h-10 µM SM 7B | 2,51E+07 | 13.212 |
| 10 µM SM 7C | 24h-10 µM SM 7C | 3,17E+07 | 13.431 |
| 10 µM SM 7D | 24h-10 µM SM 7D | 2,92E+07 | 13.378 |
| 30 µM SM 8A | 24h-30 µM SM 8A | 2,64E+07 | 13.574 |
| 30 µM SM 8B | 24h-30 µM SM 8B | 3,15E+07 | 13.787 |
| 30 µM SM 8C | 24h-30 µM SM 8C | 3,28E+07 | 13.539 |
| 30 µM SM 8D | 24h-30 µM SM 8D | 2,75E+07 | 13.608 |

**miRNA-seq quality metrics and complexity analysis**

On average, 15 x 10^6^ reads per library were generated. After an initial quality check using FastQC (Version 0.73), reads passing quality filtering were mapped to the hg38 human genome using MIRDeep2 miRNA mapper (version 2.0.1.2 ). Most reads were found to be uniquely aligned (>65% mapped reads) (Table S3). The number of miRNAs mapping to human miRBase precursors were counted using MiRDeep2 Quantifier (version 2.0.1.2). The complexity of the generated miRNA libraries was calculated as the number of detectable miRNAs (with a threshold of 10 reads/miRNA) for every million reads per sample. A total of 2,888 miRNA species mapping to miRNA precursors were identified without applying any threshold. With a threshold of 10 reads, approximately 600 mature miRNA species were captured. The numbers of generated reads and detected mature miRNAs per library are reported in Supplementary Table S4. DESeq2 was then used to normalize the read counts and to calculate differentially expressed miRNAs.

Table S3: Quality metrics for miRNA-seq. Results from MIRDeep2 miRNA mapper and quantifier (version 2.0.1.2) and SortMeRNA (Version 2.1b.6) ([Kopylova et al., 2012](#_ENREF_5)) modules are provided. Auality metrics are number of sequenced reads, percentage of reads mapped to the reference genome, and reads mapped to rRNA.

| **Sample_ID** | **Group assignment** | **Number of input reads** | **Mapped reads [%]** | **rRNA reads**  **[%]** |
| --- | --- | --- | --- | --- |
| Control 5A | 24h-untreated | 1,14E+07 | 64% | 3.00% |
| Control 5B | 24h-untreated | 8,28E+06 | 65% | 2.66% |
| Control 5C | 24h-untreated | 1,15E+07 | 64% | 3.34% |
| Control 5D | 24h-untreated | 1,11E+07 | 65% | 3.71% |
| EthOH 6A | 24h-EtOH 6A | 1,08E+07 | 68% | 3.52% |
| EthOH 6B | 24h-EtOH 6B | 1,54E+07 | 66% | 4.20% |
| EthOH 6C | 24h-EtOH 6C | 1,12E+07 | 63% | 2.64% |
| EthOH 6D | 24h-EtOH 6D | 9,05E+06 | 63% | 3.19% |
| 10 µM SM 7A | 24h-10 µM SM 7A | 1,13E+07 | 65% | 3.55% |
| 10 µM SM 7B | 24h-10 µM SM 7B | 1,50E+07 | 65% | 3.49% |
| 10 µM SM 7C | 24h-10 µM SM 7C | 1,00E+07 | 66% | 2.85% |
| 10 µM SM 7D | 24h-10 µM SM 7D | 1,37E+07 | 67% | 3.61% |
| 30 µM SM 8A | 24h-30 µM SM 8A | 1,25E+07 | 63% | 2.89% |
| 30 µM SM 8B | 24h-30 µM SM 8B | 1,44E+07 | 64% | 2.75% |
| 30 µM SM 8C | 24h-30 µM SM 8C | 1,26E+07 | 64% | 4.98% |
| 30 µM SM 8D | 24h-30 µM SM 8D | 1,11E+07 | 68% | 2.20% |

Table S4: Library complexity analysis of miRNA-seq data. The miRNA libraries'-sequence data were aligned to the human hg38 reference genome using MIRDeep2 miRNA mapper (version 2.0.1.2). MIRDeep2 miRNA quantifier (version 2.0.1.2) results were used to determine the number of miRNA species covered by at least 10 sequence reads.

| **Sample_ID** | **Group assignment** | **Number of input reads** | **Number of genes  (>10 mapped reads)** |
| --- | --- | --- | --- |
| Control 5A | 24h-untreated | 1,14E+07 | 580 |
| Control 5B | 24h-untreated | 8,28E+06 | 516 |
| Control 5C | 24h-untreated | 1,15E+07 | 571 |
| Control 5D | 24h-untreated | 1,11E+07 | 562 |
| EthOH 6A | 24h-EthOH 6A | 1,08E+07 | 574 |
| EthOH 6B | 24h-EthOH 6B | 1,54E+07 | 606 |
| EthOH 6C | 24h-EthOH 6C | 1,12E+07 | 564 |
| EthOH 6D | 24h-EthOH 6D | 9,05E+06 | 535 |
| 10 µM SM 7A | 24h-10 µM SM 7A | 1,13E+07 | 589 |
| 10 µM SM 7B | 24h-10 µM SM 7B | 1,50E+07 | 612 |
| 10 µM SM 7C | 24h-10 µM SM 7C | 1,00E+07 | 554 |
| 10 µM SM 7D | 24h-10 µM SM 7D | 1,37E+07 | 606 |
| 30 µM SM 8A | 24h-30 µM SM 8A | 1,25E+07 | 585 |
| 30 µM SM 8B | 24h-30 µM SM 8B | 1,44E+07 | 622 |
| 30 µM SM 8C | 24h-30 µM SM 8C | 1,26E+07 | 608 |
| 30 µM SM 8D | 24h-30 µM SM 8D | 1,11E+07 | 590 |

**Supplementary references**

Afgan E, Baker D, van den Beek M, Blankenberg D, Bouvier D, Čech M, Chilton J, Clements D, Coraor N, Eberhard C, Grüning B, Guerler A, Hillman-Jackson J, Von Kuster G, Rasche E, Soranzo N, Turaga N, Taylor J, Nekrutenko A, Goecks J (2016) The Galaxy platform for accessible, reproducible and collaborative biomedical analyses: 2016 update. *Nucleic Acids Res* 44: W3-w10

Anders S, Pyl PT, Huber W (2015) HTSeq--a Python framework to work with high-throughput sequencing data. *Bioinformatics (Oxford, England)* 31: 166-9

Dobin A, Davis CA, Schlesinger F, Drenkow J, Zaleski C, Jha S, Batut P, Chaisson M, Gingeras TR (2013) STAR: ultrafast universal RNA-seq aligner. *Bioinformatics (Oxford, England)* 29: 15-21

Friedländer MR, Mackowiak SD, Li N, Chen W, Rajewsky N (2012) miRDeep2 accurately identifies known and hundreds of novel microRNA genes in seven animal clades. *Nucleic Acids Res* 40: 37-52

Kopylova E, Noé L, Touzet H (2012) SortMeRNA: fast and accurate filtering of ribosomal RNAs in metatranscriptomic data. *Bioinformatics (Oxford, England)* 28: 3211-7

Love MI, Huber W, Anders S (2014) Moderated estimation of fold change and dispersion for RNA-seq data with DESeq2. *Genome biology* 15: 550

Mackowiak SD (2011) Identification of novel and known miRNAs in deep-sequencing data with miRDeep2. *Current protocols in bioinformatics* Chapter 12: 12.10.1-12.10.15
